# Myiasis in European hedgehogs (*Erinaceus europaeus*)

**DOI:** 10.1080/01652176.2025.2463328

**Published:** 2025-02-10

**Authors:** Karolin Schütte, Andrea Springer, Florian Brandes, Maximilian Reuschel, Michael Fehr, Christina Strube

**Affiliations:** a Institute for Parasitology, Centre for Infection Medicine, University of Veterinary Medicine Hannover, Hannover, Germany; b Wildlife Rescue and Conservation Center Sachsenhagen, Sachsenhagen, Germany; c Department of Small Mammal, Reptile and Avian Diseases, University of Veterinary Medicine Hanover, Hannover, Germany

**Keywords:** Strike, fly strike, blowfly, diptera, maggots, *Lucilia sericata*, *Lucilia ampullacea*, *Lucilia caesar*, *Calliphora vicina*, animal welfare

## Abstract

Myiasis due to parasitic fly larvae (maggots) can have major consequences for animal health and welfare. The European hedgehog *Erinaceus europaeus* is frequently presented in rehabilitation centres and veterinary practices due to health problems, including myiasis. In the present study, 557 hedgehogs presented at wildlife rehabilitation centres in Northern Germany during 2018–2021 were examined for the presence of dipteran eggs and larvae. Overall, 15.6% of animals carried fly eggs and/or larvae. Four different dipteran species were identified by PCR and sequencing of the internal transcribed spacer 2 (ITS-2) region. *Lucilia sericata* was detected on 25.3% [22/87] of affected hedgehogs, followed by *Calliphora vicina* (12.6% [11/87]), *Lucilia ampullacea* (11.5% [10/87]) and *Lucilia caesar* (9.2% [8/87]). Myiasis prevalence was significantly higher during the summer compared to spring and autumn. Fly eggs were found all over the body, while larvae were detected most frequently in the body’s natural orifices and in wounds. Regarding rehabilitation success, myiasis occurred significantly more frequently in animals that died or were euthanized compared to those released back into the wild. Although the high death rate probably arose in combination with underlying disease, this illustrates that myiasis represents a serious health issue that should be diagnosed and treated immediately.

## GRAPHICAL ABSTRACT

**Figure:**
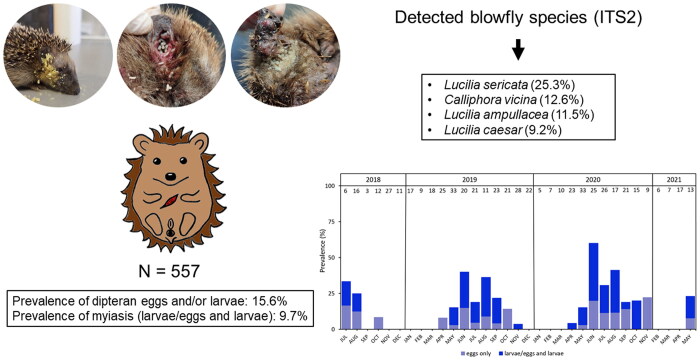

## Introduction

Myiasis, the infestation of living vertebrates with larvae (maggots) of dipteran species (Stevens and Wall 1997; Francesconi and Lupi 2012), is of major importance with regard to animal health and welfare. Among domestic animals, especially sheep, goats and rabbits are frequently affected by myiasis agents such as the blowflies *Lucilia sericata* and *Calliphora vicina*, which are of major concern for animal husbandry due to animal welfare and economic reasons (Cousquer 2006; Sotiraki and Hall 2012; Scholl et al. 2019).

Myiasis can be classified according to the anatomical location of the infestation such as aural, nasal or wound myiasis (Francesconi and Lupi 2012). Typical infestation sites are eyes, ears, mouth, nostrils, anus and genital openings as well as wounds in case of injured animals (Bexton and Couper 2019). Causative agents of myiasis can be classified into obligatory or facultative parasites (Francesconi and Lupi 2012). Species which are dependent on the host to finish their life cycle, are considered specific or obligatory myiasis agents. In wildlife, obligatory myiasis occurs for example in amphibians, with up to 70% of toads and frogs in Germany infested with *Lucilia bufonivora*, a specialised dipteran species whose larvae infest the nostrils of their host (Weddeling and Kordges 2008). Facultative myiasis agents, on the other hand, are not dependent on the host for completing their life cycle and are divided further into primary, secondary and tertiary myiasis agents. Primary myiasis agents are able to initiate myiasis in a healthy host by damaging the intact skin (Scholl et al. 2019), while secondary and tertiary agents rely on pre-damage of the skin, e.g. due to wounds or an infestation with a primary myiasis agent, or the host being near death in case of tertiary myiasis (Francesconi and Lupi 2012). Facultative myiasis is seen in many wild animal species with underlying health problems and injuries (e.g. Araghi et al. 2014; Pezzi et al. 2021). Moreover, an accidental infestation with dipteran larvae of a species which is not able to complete its life cycle on the host is referred to as accidental or pseudomyiasis (Francesconi and Lupi 2012).

Due to their urban lifestyle, European hedgehogs (*Erinaceus europaeus*) are frequently taken into human care (Lukešová et al. 2021; Bearman-Brown and Baker 2022) and are by far the most common mammalian wildlife species presented at veterinary practices (Barnes and Farnworth 2017) and wildlife rehabilitation centres in Europe (Molina-López et al. 2017; Mullineaux and Pawson 2023), including one of the centres of the present study (Wildtier- und Artenschutzstation e.V. 2021). Thus, knowledge on hedgehog diseases and their clinical management is important for veterinary practitioners. Besides many others, myiasis is one reason why hedgehogs are often presented at veterinary practices and rehabilitation centres. Frequent sites of infestation in hedgehogs seem to be the eyes and ears, although this pattern was described based on only a small number of 21 hedgehogs (Nielsen et al. 1978). Myiasis may result in the death of the animal, due to direct effects of the maggots or predisposition to predation or disease (Scholl et al. 2019). Therefore it is a serious issue in the rehabilitation of hedgehogs.

The aim of the present study was to shed light on the frequency of myiasis in European hedgehogs presented at wildlife rehabilitation centres, the infesting dipteran species, anatomical locations, seasonal infestation patterns and correlations with rehabilitation success.

## Materials and methods

### Study animals and sample collection

European hedgehogs (*n* = 557) presented at three rehabilitation centres, located in the federal state of Lower Saxony, northern Germany, were examined by a veterinarian for dipteran eggs and larvae from July 2018 until May 2021, excluding December 2020 and January 2021. Fly eggs and/or larvae were sampled *via* the use of forceps or a flea comb. Samples were then stored at −80 °C until further processing. The animals were weighed upon arrival and their age class was estimated as previously described (Schütte et al. 2024). Information on the origin of the animals was noted if available, and was characterized as urban, suburban or rural for those animals with successful differentiation of the fly species. According to their health status, the animals were later released back into the wild, euthanized or died during the rehabilitation process.

### Molecular identification of fly eggs and/or larvae

For molecular identification of the infesting fly species, a total of 69 individual samples from 60 of 87 affected animals were available, whereas no samples were available from 27 animals (Table 1). Fly egg samples were examined from 37 animals, larval samples from 31 animals and a mixed egg and larvae sample from one animal. In the case of fly eggs and small larvae, pooled samples containing several eggs or larvae were analysed, whereas large larvae were examined individually. Initially, genomic DNA isolation was performed *via* the DirectPCR Lysis Reagent (DirectPCR^®^ Cell Lysis Reagent; Peqlab, Erlangen, Germany). To 5 mg fly eggs, 100 µl of the lysis reagent and 11.1 µl Proteinase K (20 mg/ml; Macherey-Nagel GmbH & Co. KG, Dueren, Germany) were added. After mincing of the sample with a pestle, incubation at 55 °C for 5 h and inactivation of proteinase K at 85 °C for 45 min followed. The samples were centrifuged at 11.000 *g* for 1 min and the supernatant was frozen at −20 °C. As this DNA isolation technique was only successful for two samples, the remaining samples were isolated *via* NucleoSpin Tissue Kit (Macherey-Nagel GmbH & Co. KG, Dueren, Germany). Approximately 25 mg of the samples (depending on the size of the samples, min: 1 mg) were processed according to the instructions of the manufacturer using the standard protocol for human or animal tissue including proteinase K treatment, except that samples were mechanically disrupted by grinding with a pestle after adding buffer T1 and that DNA was eluted twice using 50 µl preheated (70 °C) double-distilled water each.

**Table 1.** Available number of samples and blowfly species among dipteran eggs and/or larvae from European hedgehogs (*n* = 87).

	No. of infested hedgehogs	No. of available samples	No. of successfully differentiated samples	Identified blowfly species
Infestation with dipteran eggs only	33	22	19	*Lucilia sericata* (7) *Lucilia ampullacea* (4) *Lucilia caesar* (4) *Calliphora vicina* (4)
Infestation with dipteran larvae only	28	17	12	*Lucilia sericata* (9) *Lucilia ampullacea* (2) *Lucilia caesar* (1)
Infestation with dipteran eggs and larvae	26			
Only eggs sampled		6	4	*Lucilia sericata* (1) *Lucilia ampullacea* (2) *Calliphora vicina* (1)
Only larvae sampled		5	5	*Lucilia sericata* (1) *Lucilia caesar* (1) *Calliphora vicina* (3)
Mixed egg and larvae sample		1	1	*Lucilia sericata* (1)
Eggs and larvae sampled separately^a^		18	12	
Eggs		9	6	*Lucilia sericata* (1) *Lucilia caesar* (2)^a^ *Calliphora vicina* (3)^a^
Larvae		9	6	*Lucilia sericata* (3)^a^ *Lucilia ampullacea* (2)^a^ *Calliphora vicina* (1)

^a^ For four hedgehogs, both a separate egg and larvae sample was successfully differentiated, revealing coinfestations in two individuals (*L. sericata* larvae + *C. vicina* eggs and *L. ampullacea* larvae + *L. caesar* eggs).

Species identification of fly eggs and larvae was carried out *via* amplification and sequencing of a 450 bp fragment of the internal transcribed spacer 2 (ITS-2) region with the primers ITS4 and ITS5.8 (Yusseff-Vanegas and Agnarsson 2017). The PCR reaction contained 2.5 U (0.5 µl) DreamTaq polymerase, 5 µl 10× DreamTag buffer (including 20 mM MgCl_2_; Thermo Fisher Scientific Inc., Schwerte, Germany), 1.0 µl PCR nucleotide mix (0.2 mM each; Roti^®^-Mix PCR 3, Carl Roth GmbH + Co. KG, Karlsruhe, Germany), 1.0 µl of each primer (0.2 µM final concentration) and 1 µl template DNA in a final volume of 50 µl. Cycling conditions included initial denaturation at 95 °C for 3 min, 38 cycles of denaturation at 95 °C for 30 s, annealing at 44 °C for 30 s and extension at 72 °C for 1 min, followed by final extension at 72 °C for 7 min. For visualisation, a 1.5% agarose gel supplemented with GelRed^®^ (1:10,000; Biotium, Inc., Fremont, CA, USA) was used. Custom Sanger sequencing of PCR products was carried out at Microsynth Seqlab Laboratories (Göttingen, Germany). The obtained sequences were BLASTed against the NCBI database.

### Statistical analysis

Statistical analyses were conducted in R v. 4.1.0. (R Core Team 2016). Absence/presence data of fly larvae in relation to sampling season, sampling year, animal sex, age class, bodyweight and animal survival was investigated using a binomial generalized linear model (GLM). Due to missing data, the model was calculated for a subset of 431 animals. The model was compared to a null model containing only the intercept in a likelihood ratio test (R function anova, test = ‘chisq’). Multiple comparisons between the different seasons *via* Tukey contrasts were conducted *via* the R-function ‘glht’ (package ‘multcomp’). The survival rate of animals infested with fly eggs only and with fly larvae was compared *via* Chi-Square test.

## Results

### Myiasis prevalence and fly species identification

From a total of 557 hedgehogs examined, 15.6% (87/557) carried fly eggs and/or larvae. While 5.9% (33/557) of animals carried eggs only, 5.0% (28/557) were infested with larvae only, and 4.7% (26/557) with both eggs and larvae. This equates to 10.6% (59/557) of hedgehogs carrying fly eggs, and a myiasis prevalence (infestation with larvae) of 9.7% (54/557).

Molecular species differentiation was successful for 53 samples (29 egg samples, 23 larvae samples, 1 mixed egg/larvae sample) from 49 animals. Detailed information on successful differentiation regarding the infestation type (eggs, larvae or mixed) is provided in Table 1, and frequencies with regard to fly species are listed in Table 2. Overall, four different dipteran species of the genera *Lucilia* and *Calliphora* were identified (GenBank accession numbers PP488225-PP488276, PP489910). *Lucilia sericata* was the most frequently identified species, followed by *C. vicina*, *Lucilia ampullacea* and *Lucilia caesar*. While the species distribution in dipteran egg samples was almost equal, more than every second larvae sample was differentiated as *L. sericata* (Table 2). From nine of 26 hedgehogs carrying both fly eggs and larvae, separate egg and larvae samples were available. Unfortunately, in four cases only the eggs (two samples) or larvae (two samples) could be successfully differentiated, and from one animal neither sample could be identified. Of the remaining four animals, both the egg and larvae samples were successfully differentiated. Two of these hedgehogs carried eggs and larvae of the same species, *C. vicina* in one case and *L. sericata* in the other. In the other two animals, coinfestation with two species was observed. One animal carried larvae of *L. sericata* and eggs of *C. vicina*, the second hedgehog was infested with larvae of *L. ampullacea* and eggs of *L. caesar*. The origin (urban, suburban or rural area) of hedgehogs with successfully differentiated fly species is shown in Table 3.

**Table 2.** Distribution of blowfly species among successfully differentiated samples from European hedgehogs (*n* = 49).

	No. of samples (% of differentiated samples)			Positive hedgehogs (% of hedgehogs with differentiated samples)
	Dipteran eggs (*n* = 29)	Dipteran larvae (*n* = 23)	Mixed egg and larvae sample (*n* = 1)	
*Lucilia sericata*	9 (31.0%)	13 (56.5%)	1 (100%)	22 (44.9%)
*Lucilia ampullacea*	6 (20.7%)	4 (17.4%)	0 (0.0%)	10 (20.4%)
*Lucilia caesar*	6 (20.7%)	2 (8.7%)	0 (0.0%)	8 (16.3%)
*Calliphora vicina*	8 (27.6%)	4 (17.4%)	0 (0.0%)	11 (22.4%)

**Table 3.** Origin of 49 European hedgehogs with differentiated fly species.

Species^a^	No. of hedgehogs infested	Urban origin (%)	Suburban origin (%)	Rural origin (%)	Unknown origin (%)
*Lucilia sericata*	22	7 (31.8%)	2 (9.1%)	7 (31.8%)	6 (27.3%)
*Lucilia ampullacea*	10	4 (40.0%)	3 (30.0%)	1 (10.0%)	2 (20.0%)
*Lucilia caesar*	8	1 (12.5%)	2 (25.0%)	2 (25.0%)	3 (37.5%)
*Calliphora vicina*	11	6 (54.5%)	1 (9.1%)	1 (9.1%)	3 (27.3%)

^a^ One coinfected hedgehog with *Lucilia sericata* and *Calliphora vicina* had an urban origin; one coinfected hedgehog with *Lucilia ampullacea* and *Lucilia caesar* had a suburban origin.

### Affected body localisations

**Figure 1.**
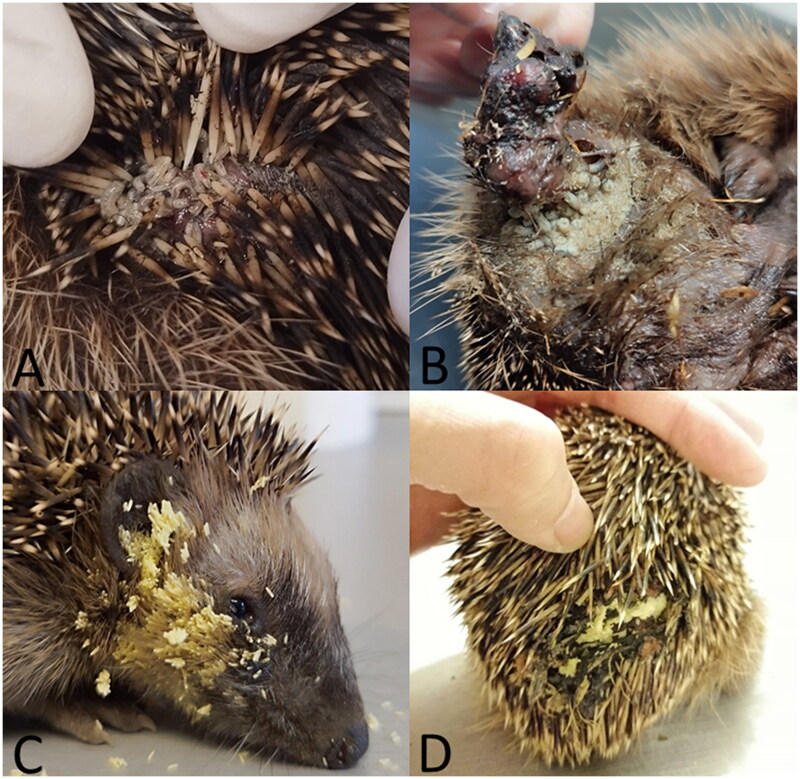
Infestation with dipteran larvae of the body surface (A) and wounds (B) as well as presence of dipteran eggs on the head (C) and in wounds (D) of European hedgehogs.

**Figure 2.**
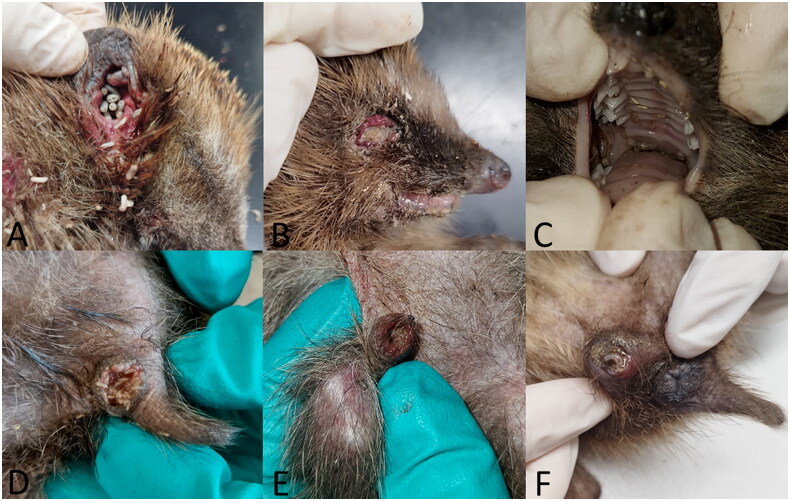
Infestation of natural body orifices (A: ear, B: eye, C: oral cavity, D: anus, E: preputium, F: vulva) with dipteran larvae in European hedgehogs.

**Figure 3.**
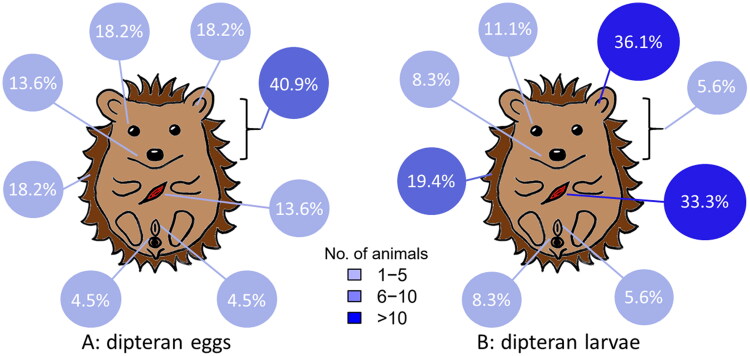
Distribution of affected body areas among 22 European hedgehogs carrying dipteran eggs (A) and 36 hedgehogs carrying dipteran larvae (B). Body localisations are (clockwise starting at the bottom left): anus, body surface, oral cavity, eye, ear, head (bracket), wound and genital opening. Blue shading and size of the circles indicate the absolute numbers of affected individuals, while percentage values refer to the detection frequency among the total number of infested animals.

Fly eggs and larvae were found all over the hedgehogs’ body surface and wounds (Figure 1) as well as its natural orifices (Figure 2). The localisation of eggs was documented in 22 (37.3%) of 59 cases, with some animals carrying eggs on multiple body parts. Eggs were seen predominantly on the body surface (59.1% [13/22]), precisely on the head (9), abdomen (2), back (1) and unspecified anatomical location (1), followed by body orifices (40.9% [9/22]) and wounds (13.6% [3/22]). The localisation of larvae was noted in 36 (67.7%) of 54 animals, again including animals with multiple affected body parts. Larvae were found most often in the body’s natural orifices (50.0% (18/36]), especially the ears (36.1% [13/36]), and in wounds (33.3% [12/36]) followed by the body surface (25.0% [9/36]), precisely on the head (2), abdomen (1), back (1), inner thigh (1), chest (1), right side of the body (1) and unspecified location (2). The localisations of eggs and larvae are graphically summarised in Figure 3.

Regarding the localisation of the different dipteran species, all four detected species were found on the body surface, *L. sericata* and *C. vicina* were detected in the body’s natural orifices and all three *Lucilia* spp. were found in wounds (Table 4).

**Table 4.** Recorded localisation of different dipteran species in European hedgehogs infested with eggs (22 animals) and larvae (36 animals).

	Eggs			Larvae		
Species	Head/body surface	Orifices	Wounds	Head/body surface	Orifices	Wounds
*Lucilia sericata*	4	4	0	2	3	5
*Lucilia ampullacea*	1	0	0	1	0	2
*Lucilia caesar*	2	0	2	0	0	0
*Calliphora vicina*	1	3	0	1	2	0
n.d.	5	2	1	5	13	5

n.d. = not determined.

### Predisposing factors and association with rehabilitation success

With 90.8% (79/87), almost all affected hedgehogs were adult, followed by 9.2% (8/87) subadults, while the age distribution in the non-affected hedgehogs was 58.3% (274/470) adults, 40.2% (189/470) subadults and the age of 1.5% (7/470) was not determined. However, the statistical analysis indicated no significant difference in myiasis prevalence between the age classes, nor between the sexes. Furthermore, no correlation with animal weight was observed (Table 5). Health issues in addition to the presence of fly eggs and/or larvae were mainly injuries with an open wound (33.3% [29/87]), whereby the presence of fly stages in these wounds was only noted in 15 cases, followed by general weakness (21.8% [19/87]), respiratory problems (14.9% [13/87]), traumatic injuries without an open wound (paralysis of hindlimbs [2], contusion due to snap traps [2], swollen head [1], fractured skull [1], healed limb fracture [1], stiff limb [1], hip fracture [1], hematoma [1], joint luxation [1]; 12.6% [11/87]), having been trapped in a net or cellar shaft (5.7% [5/87]), abscesses (2.3% [2/87]), neoplasia (2.3% [2/87]) and one case each of diarrhea, dermatitis and mite infestation (1.1% [1/87]), while no problem apart from the infestation with fly eggs and/or larvae was documented in the remaining animals (3.4% [3/87]).

**Figure 4.**
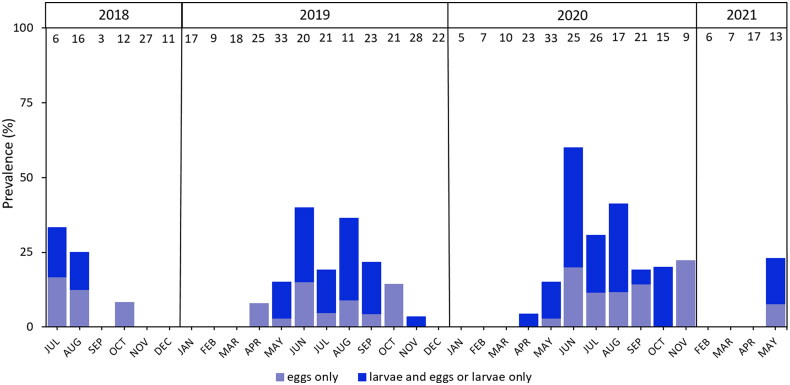
Detection frequency of dipteran eggs and/or larvae on European hedgehogs in Northern Germany during the years 2018–2021. Total numbers of examined animals per month are shown above the bars. No animals were examined in the months December 2020 and January 2021. Note that 26 (4.7%) of the 557 hedgehogs were infested with both eggs and larvae.

The myiasis prevalence showed a statistically significant peak during the summer months with the highest prevalence in June 2019 and 2020 (Table 5; Figure 4), whereas no cases of myiasis were recorded between December and March.

**Table 5.** Results of the binomial GLM testing the association of dipteran larvae infestation (myiasis) in European hedgehogs with sampling season, sampling year, sex, age, weight and the animal’s outcome.

	Est.	SE	z	*p*
Intercept	−3.55	0.75	−4.73	**<0.001**
Season^a^				
Summer vs. spring	1.62	0.49	3.33	**0.003**
Autumn vs. spring	0.33	0.56	0.59	0.921
Winter vs. spring	−14.98	837.54	−0.02	1.000
Autumn vs. summer	−1.29	0.48	−2.71	**0.025**
Winter vs. summer	−16.60	837.54	−0.02	1.000
Winter vs. autumn	−15.31	837.54	−0.02	1.000
Sampling Year (ref.: 2018)				
2019	0.79	0.72	1.10	0.271
2020	0.86	0.69	1.24	0.214
2021	0.62	1.09	0.57	0.571
Sex (ref.: Male)				
Female	−0.16	0.35	−0.47	0.642
Age (ref.: Subadult)				
Adult	1.25	0.73	1.71	0.087
Weight	0.00	0.00	−1.29	0.197
Outcome (ref.: Released)				
Euthanised	1.36	0.41	3.31	**0.001**
Died	1.48	0.47	3.13	**0.002**

Est. = Estimate; SE = Standard Error. ^a^Multiple comparisons between the different seasons were conducted *via* the R-function ‘glht’ (package ‘multcomp’). The model was significantly different from a null model (Chi-square = 85.48, Df = 11, *p* < 0.001). Statistically significant *p*-values (*p* ≤ 0.05) are shown in bold.

Regarding the fate of the animals, myiasis prevalence was significantly higher in animals which died or had to be euthanized (Table 5). Among animals with myiasis, 29 (53.7%) individuals had to be euthanized, 13 (24.1%) hedgehogs died and 12 (22.2%) were released back into the wild. Especially animals with open wounds had to be euthanized often (72.4%, [21/29]). No significant difference in the survival of hedgehogs carrying fly eggs only (15.2% [5/33]) versus hedgehogs carrying larvae or eggs and larvae (22.2% [12/54]) was determined (Chi-square = 0.28, Df = 1, *p* = 0.597). The rehabilitation success in relation to the detected fly species is shown in Table 6.

**Table 6.** Rehabilitation success of 49 European hedgehogs with differentiated fly species.

Species^a^	No. of infested hedgehogs	No. of deceased hedgehogs (%)	No. of released hedgehogs (%)
*Lucilia sericata*	22	20 (90.9%)	2 (9.1%)
*Lucilia ampullacea*	10	8 (80.0%)	2 (20.0%)
*Lucilia caesar*	8	6 (75.0%)	2 (25.0%)
*Calliphora vicina*	11	8 (72.7%)	3 (27.3%)

^a^ One hedgehog coinfected with *Lucilia sericata* and *Calliphora vicina* and one hedgehog coinfected with *Lucilia ampullacea* and *Lucilia caesar* were euthananized.

## Discussion

In this large dataset of 557 hedgehogs presented at wildlife rehabilitation centres, more than one-tenth of hedgehogs carried fly eggs and/or larvae, with 9.7% (54/557) suffering from myiasis as indicated by the presence of larvae. Although no information on the frequency of myiasis in the healthy, free-ranging hedgehog population is available, this illustrates that at least those hedgehogs taken into human care are frequently affected.

Typical dipteran species responsible for myiasis in hedgehogs are *Lucilia* spp. and *Calliphora* spp. (Robinson and Routh 1999; Heinrich 2006; Bexton 2016). Nielsen et al. (1978) described oviposition of the blowflies *C. vicina*, *L. ampullacea*, *L. caesar*, *Lucilia illustris* and *Sarcophaga melanura* on hedgehogs. In the present study, the most frequently identified blowfly species was *L. sericata*. This species is a primary myiasis agent with a high pathogenicity (Stevens and Wall 1997; Francesconi and Lupi 2012). In contrast, *L. caesar*, *L. ampullacea* and *C. vicina*, which were also identified in the present study, are usually considered facultative secondary ectoparasites (Stevens and Wall 1997) and thus need an underlying predisposition to harm the host. However, Nielsen et al. (1978) described *C. vicina* and *L. ampullacea* as primary myiasis agents in hedgehogs, as no coinfesting species were found. Moreover, Francesconi and Lupi (2012) refer to *C. vicina* as a potential primary myiasis agent. In the present study, the four animals which carried *C. vicina* larvae showed no open wounds and *C. vicina* was found on the head of the host as well as in the body’s natural orifices, which suggests that this species might indeed act as a primary myiasis agent, although the affected hedgehogs had other health problems such as apathy, hypothermia and emaciation. However, as the larval developmental stage was unfortunately not documented, it is not possible to say whether they were more or less freshly hatched first-stage larvae or whether they were able to develop into the second or third larval stage, which would actually indicate primary myiasis.

Almost half of the animals infested by *L. ampullacea* had an open wound (4), other affected animals showed apathy (2), respiratory problems (2) and an abscess with dermatophyte infection (1). Only one animal which carried larvae of *L. ampullacea* had no open wound, the remaining four animals without an open wound carried only eggs of *L. ampullacea*, i.e. did not develop myiasis at least until the time of examination. Furthermore, it should be kept in mind that only a random sample of larvae and eggs was examined per animal, so it cannot be excluded that other dipteran species were involved in these cases. Conclusively, the aspect that *C. vicina* and *L. ampullacea* might act as primary myiasis agents should be investigated further.

Coinfestation with *L. sericata* and *C. vicina* was observed in one animal. Since larvae were determined as *L. sericata* and eggs as *C. vicina*, it is most likely that *L. sericata* initiated myiasis and *C. vicina* followed as a secondary myiasis pathogen. The second case of coinfestation included *L. ampullacea* larvae and *L. caesar* eggs. This animal had a big open wound of probable traumatic origin, conclusively both species can be regarded as secondary myiasis agents in this case. Similar coinfestations with up to four different species were described by Nielsen et al. (1978).

Blowfly larvae were frequently observed in the body’s natural orifices, with the ear as the predominant infestation site, as well as in open wounds. Similarly, Nielsen et al. (1978) observed larvae most often in the natural body’s orifices, keeping in mind the small sample size of only 21 animals in that study. In contrast, fly eggs were often attached to the body surface and the head in the present study. This shows that flies position their eggs all over the body, while fly larvae were not evenly distributed. One hypothesis for larvae being localized mainly in the body’s natural orifices and wounds could be that those larvae which hatch on other parts of the body are not able to survive and develop further due to a lack of food. This might be the case especially for secondary myiasis agents, and is supported by the fact that the proportion of the primary agent *L. sericata* was higher among the differentiated larvae than among differentiated egg samples, indicating that larvae of secondary species may not have survived after hatching. Alternatively, larvae could have actively moved to body locations such as the orifices or wounds after hatching, which has also been proposed by Rivers et al. (2023). Directed movement of fly larvae was shown in studies on maggot mass aggregation and temperature selection (Johnson et al. 2014) as well as in studies on the post-feeding dispersal away from the host for pupation (Gomes et al. 2006). The movement behaviour of first-stage larvae requires further investigation in order to better understand the exact pathogenesis of myiasis for different blowfly species. Regarding the infestation localisation of the different species, only eggs and larvae of *L. sericata* and *C. vicina* were detected in the body’s natural orifices, but not *L. ampullacea* nor *L. caesar*, which were located on the intact body surface as well as in wounds. Especially for primary myiasis agents, the orifices are an easy way to enter the host’s body.

Regarding the origin of infested animals, hedgehogs carrying *L. sericata* were found in urban and rural areas likewise, but less often in suburban areas. *Lucilia ampullacea* occurrence declined from urban to rural areas and *C. vicina* most often originated from urban areas, possible being attracted more by humans or waste dumps than by animals. In contrast, animals infested by *L. caesar* were found more often in suburban and rural areas. However, due to the small number of animals this aspect has to be treated with caution and needs further investigation.

As expected, the myiasis prevalence was significantly higher in summer than in the colder seasons, as most of the detected dipteran species are not active during the winter months. Only *C. vicina* is known to show a year-round activity in Europe, including Germany (Zabala et al. 2014; Lutz et al. 2022). In addition, also the study animal species itself shows seasonal changes in activity. Due to hibernation in winter, fewer hedgehogs were examined during this season, lowering the probability of detecting myiasis cases in the colder seasons. Furthermore, as blowflies are usually active during the day (Amendt et al. 2008), healthy hedgehogs are less at risk of becoming infested as they display predominantly nocturnal activity and spend the day in their nests. However, weak or sick hedgehogs may show diurnal activity or may rest in the open without an adequate nest, as reported by the citizens finding the animals. This may result in an increased risk of blowfly oviposition. Furthermore, the summer daytime nests of hedgehogs are not as firmly built as the hibernating nests and sometimes only consist of a sheltered place in the vegetation (Morris 1973; Reeve and Morris 1985), resulting in the possibility of flies entering the nest, also infesting healthy individuals. Additionally, nocturnal oviposition by blowflies has been observed under laboratory conditions (Amendt et al. 2008). Thus, it cannot be excluded that hedgehogs also become infested at night.

Although no statistically significant effect of age was observed, most hedgehogs suffering of myiasis in the recent study were adult individuals (90.8%). However, as only subadult and adult but no juvenile hedgehogs were examined, the age distribution might be biased. Nevertheless, an explanation for a higher prevalence in adults could be the fact that adult hedgehogs are often presented in a worse clinical condition compared to juvenile hedgehogs, which are often simply considered orphaned, but have no acute health problem (Garcês et al. 2020).

All affected hedgehogs showed clinical signs of health impairment. Whether this health impairment was the cause or the consequence of myiasis remains inconclusive in some cases. However, every third (33.3% [29/87]) infested animal had an open wound, thus, injured hedgehogs seem to be at the highest risk of myiasis. In half of the cases, the open wound could be attributed to a traumatic cause such as cuts, possibly from gardening tools, and fractures presumably caused by traffic accidents. The remaining 15 hedgehogs had injuries of unknown origin. These injuries may therefore also have been caused by the damaging effect of the larvae. Overall, only 22.2% (12/54) of animals infested with larvae survived. In comparison, 68.1% (320/470) of non-infested animals could be released back into the wild. But since there was no difference between the survival rate of animals infested with eggs only and animals infested with larvae, it is probably not the myiasis alone that is responsible for the high death rate but rather an underlying disease combined with the subsequent myiasis. Looking at the infestation with the different fly species separately, *L. sericata* was more often associated with death of the host (90.9%) than the other detected fly species (72.7–80.0%), indicating that it may be the most pathogenic species.

In conclusion, it is important to carefully examine hedgehog patients for fly eggs and larvae and treat affected animals as soon as possible to prevent further damage (Bexton and Couper 2019). Bexton (2016) recommends manual removal of eggs and larvae and the use of topical cyromazine, insecticidal ear drops, topical spot on solutions (e.g. permethrin, ivermectin) or injectable ivermectin in addition to fluid therapy, antibiotics, and non-steroidal anti-inflammatory drugs (NSAIDs). For systemic treatment, doramectin, nitenpyram (Wrobbel 2011) or isoxazolines, e.g. fluralaner (Ribeiro Campos et al. 2021), can also be used. Especially regarding long-acting molecules a negative effect on the ecosystem needs to be considered as insecticidal drugs might also affect non-target organisms such as dung beetles (Suárez et al., 2009). However, the main excretion of macrocyclic lactones, for example, takes place during the first days after treatment, as shown in a study on horses where 90% of ivermectin was excreted after four days and of moxidectin after eight days, respectively (Pérez et al., 2001). Environmental contamination by treated hedgehogs can be avoided by not releasing hedgehogs into the wild immediately after antiparasitic treatment and by disposing hedgehog faeces *via* household waste. In case of systemic treatment, it should always be kept in mind that weak hedgehogs are especially sensitive to intoxication and should be stabilised before drug administration. Additionally, nitenpyram tablets can also be dissolved and used topically to flush wounds in order to kill larvae (Polak et al. 2018), but care should be taken not to dilute the solution too much to retain the lethal effect on larvae. When housing hedgehogs, it is important to prevent myiasis by using fly nets and paying particular attention to hygiene. A fast diagnosis and treatment can be lifesaving in rescue hedgehogs and is important to protect animal health and welfare, but in severe cases euthanasia may need to be considered.

## Data Availability

Data supporting reported results is contained within the article. Generated sequences were deposited in GenBank under accession nos. PP488225-PP488276 and PP489910.

## Abbreviations

BLAST: Basic Local Alignment Search Tool
Df: degrees of freedom
GLM: generalized linear model
ITS-2: internal transcribed spacer 2
NCBI: National Centre for Biotechnology Information
PCR: polymerase chain reaction
SD: standard deviation
SE: standard error
